# Final adult height of children with idiopathic short stature: a multicenter study on GH therapy alone started during peri-puberty

**DOI:** 10.1186/s12887-020-02034-8

**Published:** 2020-03-28

**Authors:** Di Wu, Rui-min Chen, Shao-ke Chen, Ge-li Liu, Lin-qi Chen, Yu Yang, Xin-li Wang, Ya-guang Peng, Chun-xiu Gong

**Affiliations:** 1 Department of Endocrine and Genetics and Metabolism, Beijing Children’s Hospital, Capital Medical University, National Centre for Children’s Health, No. 56 Nanlishi Road, Xicheng District, Beijing, 100045 China; 2 Department of Endocrinology, Fuzhou Children’s Hospital of Fujian Medical University Teaching Hospital, No.145, 817 Middle Road, Gulou District, Fuzhou, Fuzhou, 350005 Fujian Province China; 3 Department of Pediatric, The second Affiliated Hospital of Guangxi Medical University, No.166, Daxuedong Road, Nanning, Guangxi, Nanning, 530007 Guangxi China; 4 Department of Pediatric, Tianjin Medical University General Hospital, No.154, Anshan Road, Heping District, Tianjin, 300052 Tianjin China; 5 Depatment of Endocrinology, Children’s Hospital of Soochow University, No. 92, Zhongnan Street, Gongyeyuan District, Suzhou, Suzhou, 215025 Jiangsu China; 6 Department of Endocrinology, Children’s Hospital of Jiangxi Province, No.122, Yangming Road, Donghu District, Nanchang, Jiangxi, Nanchang, 330006 Jiangxi China; 7 Department of Pediatric, Peking University Third Hospital, No.49, Huayuanbei Road, Haidian District, Beijing, Beijing, 100191 Beijing China; 8 Center for Clinical Epidemiology and Evidence-Based Medicine, Beijing Children’s Hospital, Capital Medical University, National Centre for Children’s Health, No.56, Nanlishi Raod, Xicheng District, Beijing, Beijing, 100045 Beijing China

**Keywords:** Final adult height (FAH), Idiopathic short stature (ISS), Standard deviation score (SDS), Baseline height, Target height (TH)

## Abstract

**Background:**

To evaluate the efficacy of GH in improving FAH in ISS children in a multicenter study.

**Methods:**

A real-world observation was carried out. Children with ISS in seven hospitals in China were enrolled. The height gains standard deviation score and the height gain over the target height were evaluated.

**Results:**

There were 344 ISS patients (217 boys and 127 girls). The baseline average age of boys and girls was 12.7 and 11.7 years, with bone age of 11.7 and 10.1 years, respectively. The baseline height SDS of boys and girls was − 3.07 and − 2.74, and the FAH SDS was − 1.91 and − 1.38, respectively. Compared with the baseline height SDS, the FAH SDS was significantly increased in both boys and girls (both *P* = 0.0000). The FAH SDS was the highest (gain by 1.54 SD) in the ≥2y treatment course group. Two hundred eighteen patients (218/344, 63.4%) had a FAH SDS > − 2 SD. Among these patients, girls in the 1-2y treatment course group and ≥ 2y group had a FAH SDS higher than TH SDS. Even in the control group, a spontaneous catch-up growth of 1.16 SD was observed. A multivariate linear regression model was used to analyze the results, with FAH SDS as the dependent variable. It was found that the treatment course and baseline height SDS in the boys’ model were statistically significant (*P* < 0.05), whereas the baseline height SDS and baseline bone age significantly affected the girls’ FAH SDS (*P* < 0.05).

**Conclusions:**

Both girls and boys of ISS improved FAH by GH therapy even if treatments begin over 10 years old and majority of them reached TH. Some peri-puberty ISS will have a spontaneous height gain. We recommend the course of GH treatment more than 2 years for girls, and longer courses for boys.

## Background

Idiopathic short status (ISS) is defined as a condition in which the height of an individual is more than 2SD score (SDS) below the corresponding mean height for a given age, sex, and population group without evidence of systemic, endocrine, nutritional, or chromosomal abnormalities. Children with ISS have normal birth weight and are GH sufficient [1]. The incidence of ISS (including constitutional delay of growth and puberty and familial short stature) is about 23 in 1000 [1–3]. In 2003, the US Food and Drug Administration approved growth hormone (GH) for the treatment of ISS patients (height < − 2.25 SD). The main purpose of GH therapy for ISS is to attain normal adult height and avoid daily life inconvenience and psychological problems caused by extreme or unacceptable short stature. However, few clinical studies have explored whether the final adult height (FAH) can reach the normal range after GH therapy. FAH is considered the golden indicator for evaluating the efficacy of GH therapy [4, 5]. However, the predicted FAH following a short period of treatment is dynamic and cannot reflect the actual FAH. Owing to the heterogeneity of the treated ISS populations and the individualization of treatment, only a few randomized trials with small sample sizes have observed ISS until FAH [6–10]. A randomized study of FAH typically takes 8 years or more to complete and is often difficult to implement in clinical settings. Thus, most of the currently available studies only have small samples and are carried out in a single center. More studies are needed to confirm the efficacy of GH in the treatment of ISS.

In the clinical real world, many ISS children’s parents are willing to observe their children growth when they are young. As children grow older and become peri-puberty, more people come to see doctors. What is the effect of GH treatment alone in peri-puberty? Past literatures are not accurate. This is the first multicenter study in China on the efficacy of growth hormone alone in the treatment of elder children. In our current study, we followed up children with ISS diagnosed by the departments of pediatric endocrinology in seven tertiary hospitals in different regions of mainland China, and evaluated the efficacy of GH for ISS children until FAH.

## Methods

### Subjects

Patients with ISS confirmed in the departments of pediatric endocrinology in seven tertiary hospitals, namely Beijing Children’s Hospital Affiliated to Capital Medical University, Fuzhou Children’s Hospital of Fujian Medical University Teaching Hospital, The second Affiliated Hospital of Guangxi Medical University, General Hospital of Tianjin Medical University, Children’s Hospital of Soochow University, Children’s Hospital of Jiangxi Province, and Third Hospital of Peking University, were enrolled in this study.

The inclusion criteria included: (a) body height less than − 2 SD of the height in the general population with the same race, age, sex, and other factors; (b) without systemic disease, endocrine disease, nutritional disease, or chromosomal abnormality; (c) with normal body length and weight at birth; (d) with a serum peak GH concentration > 7 ng/mL at peak GH stimulation test and normal insulin-like growth factor 1; (e) born before January 1, 2001; (f) had been treated with GH alone for ISS but the GH therapy had been withdrawn and FAH reached, regardless of whether the patient was in Tanner stage 1 at admission; and (g) informed consent was signed by parents and children older than 8 years.

The exclusion criteria included: (a) children who were treated with GnRHa (gonadotropin-releasing hormone agonist); and (b) obese children.

Regarding the concept of peri-puberty, there is no well-recognized age limit for its definition. Since the baseline Tanner stages differed in our research populations, the term peri-puberty was used in our study.

All patients and their parents signed informed consent for data collection.

### Methods

A real-world observation was carried out. Clinical data including name, sex, date of birth, age at baseline, baseline height, baseline bone age based on Greulich-Pyle methodology, GH treatment course, age at last follow-up, FAH, and parents’ heights were recorded. The baseline height SD score (SDS), FAH SDS, and target height (TH) SDS were calculated. GH was subcutaneously injected at a dose of 0.15–0.2 IU/kg/day.

The height SD score (HtSDS) was calculated by referring to the 2005 Standard Deviations of Height and Weight for Children and Adolescents Aged 0–18 years in China [11].

Height standard deviation score (HtSDS) = (actual height − average height for children of the same sex and age) ÷ (SD of the heights for children of the same sex and age) [11].

TH (i.e., mid-parental target height) was as follows:

$$ \mathrm{Boys}'\mathrm{TH}\ \left(\mathrm{cm}\right)=\left(\mathrm{father}'\mathrm{s}\ \mathrm{height}+\mathrm{mother}'\mathrm{s}\ \mathrm{height}+13\right)\div 2 $$

$$ \mathrm{Girls}'\mathrm{TH}\ \left(\mathrm{cm}\right)=\left(\mathrm{father}'\mathrm{s}\ \mathrm{height}+\mathrm{mother}'\mathrm{s}\ \mathrm{height}-13\right)\div {2}^{(12)} $$

FAH was defined as follows: age at the last follow-up was > 15 years, height velocity was below 1 cm/year, and the GH therapy had been discontinued [12].

The primary endpoint was the difference between FAH SDS and baseline height SDS, i.e., the height SDS gain, expressed as △1HtSDS. The secondary endpoint was the difference between FAH SDS and TH SDS, i.e., the height gain over the TH (△2HtSDS). △3HtSDS is baseline height SDS minus TH SDS. In addition, influencing factors of height SDS were analyzed.

The control group comprised patients who had been treated for less than 3 months. The remaining patients were divided into the 3-6 m group (treated for 3–6 months), 6-12 m group (treated for 6 months to 1 year), 1-2y group (treated for 1–2 years), and ≥ 2y group (treated for 2 or more years).

Patients were also stratified according to gender.

Screening flowchart: 
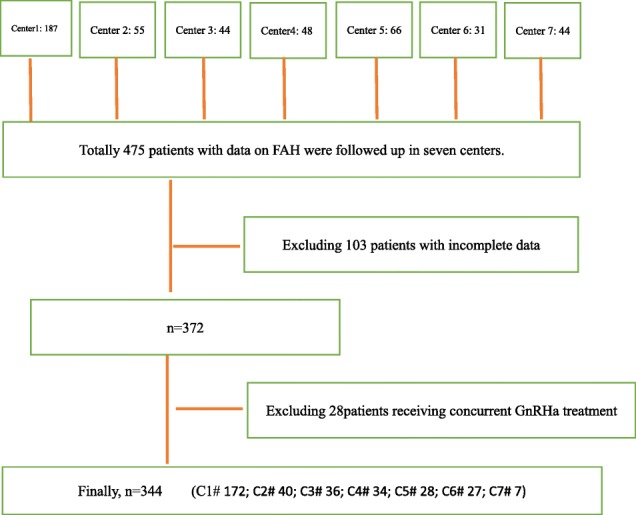


### Statistical analysis

Statistical analysis was performed using SPSS 20.0 software. A normal distribution test showed that all measurement data were normally distributed. Data are presented as mean ± SD. The means of two independent samples were compared by using the *t* test, and the comparisons of means among multiple groups were based on analysis of variance. The influencing factors of FAH SDS were analyzed by multivariate linear regression. A *P* value of less than 0.05 was considered significantly different.

## Results

### General data

Among the 344 ISS patients in seven centers, there were 217 boys and 127 girls. The average age of boys and girls when starting the treatment (baseline) was 12.7 ± 1. 87 and 11.7 ± 1.61 years, with bone age of 11.7 ± 2.32 years and 10.1 ± 2.03 years, respectively. The growth stopped at the final follow-up visit, with a mean age of 18.5 ± 2.25 years for boys and 18.0 ± 2.02 years for girls (Table 1).

**Table 1:** General data

	Boys	Girls	t	P
Baseline age (years)	12.7 ± 1.87	11.7 ± 1.61	4.741	0.000*
Baseline bone age (years)	11.73 ± 2.32	10.08 ± 2.03	−1.379	0.169
Treatment course (years)	1.33 ± 1.296 (0.06–8.33)	1.33 ± 1.159 (0.08–5.65)	−0.046	0.963
Age at the final follow-up (years)	18.5 ± 2.25	18.0 ± 2.02	1.951	0.052
Baseline body height SDS	−3.07 ± 1.054	−2.74 ± 0.761	− 3.311	0.001*
FAH (cm)	160.6 ± 8.09	153.0 ± 5.46	10.404	0.000*
FAH SDS	−1.91 ± 1.284	− 1.38 ± 1.017	−4.264	0.000*
TH (Median height of parents (cm)	167.7 ± 4.51	155.6 ± 4.12	23.354	0.000*
TH SDS	−0.83 ± 0.747	−0.98 ± 0.766	1.651	0.100
△1 (FAH SDS - baseline height SDS)	1.15 ± 1.387	1.49 ± 0.955	−2.605	0.010*
△2 (FAH SDS - TH SDS)	−1.10 ± 1.403	−0.27 ± 1.271	−5.215	0.000*
△3 (baseline height SDS - TH SDS)	−2.22 ± 1.291	−1.74 ± 0.931	−3.413	0.001*
Comparisons between FAH SDS and baseline height SDS	P = 0.000* t = 10.245	P = 0.000* t = 11.880		

The baseline height SDS of boys and girls was − 3.07 and − 2.74, and the FAH SDS was − 1.91 and − 1.38, respectively. Compared with the baseline height SDS, the FAH SDS was significantly increased in both boys and girls (both *P* = 0.0000) (Table 1).

### Comparisons between FAH SDS and baseline height SDS

According to the treatment course, patients who had been treated for less than 3 months were designated the control group, and the remaining patients were divided into the 3-6 m, 6-12 m, 1-2y, and ≥ 2y groups. The average course of treatment was 2.92 years in the ≥2y group. The baseline height SDS and TH SDS were comparable among the five groups.

The baseline ages of the control group, 3-6 m group, and 6-12 m group were significantly larger than that of the ≥2y group.

**Fig. 1:**
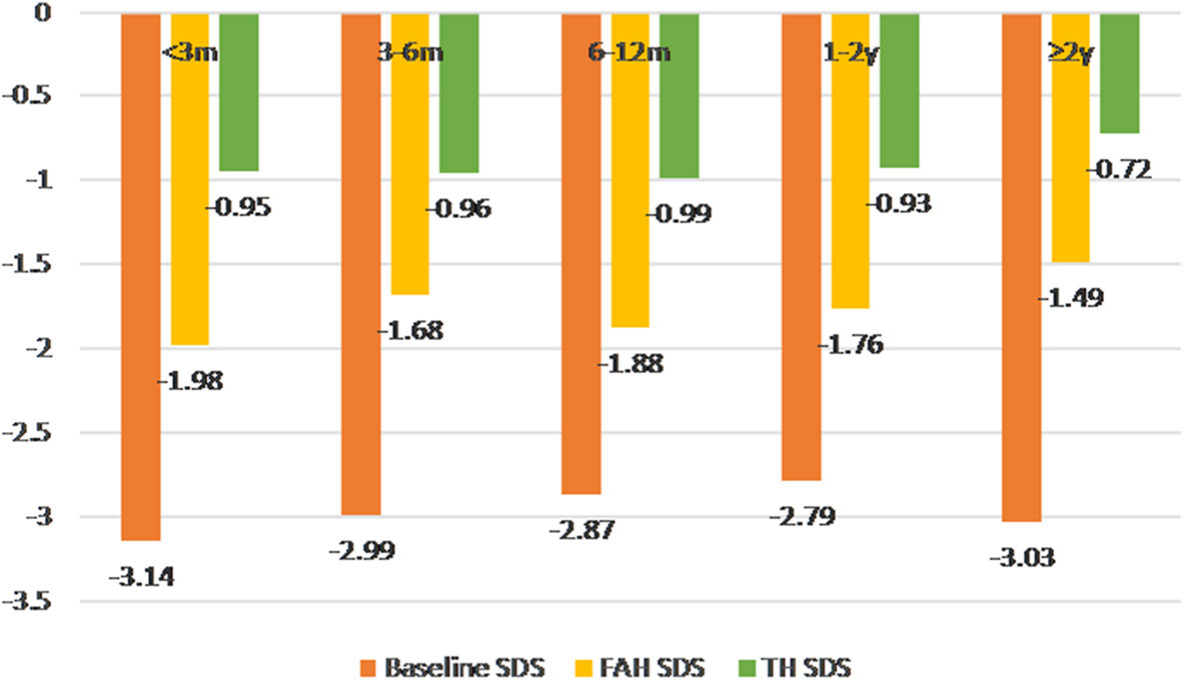
Baseline height SDS, FAH SDS, and TH SDS in each group

The height gain SDS (i.e., between FAH SDS and baseline height SDS [△1HtSDS]) was 1.16, 1.30, 1.00, 1.01, and 1.54 in each group; compared with the control group, the *P* value was 0.503, 0.492, 0.525, and 0.082, showing no significant difference. However, the FAH SDS was highest (increased by 1.54 SD) in the ≥2y group. The △1HtSDS showed a gradually increasing trend in the 6-12 m group, 1-2y group, and ≥ 2y group. Compared with the other two groups, the ≥2y group had significantly different △1HtSDS (*P* = 0.022). Even in the control group (regarded as an untreated group), a spontaneous catch-up growth of 1.16 SD was observed. However, the absolute height gain (△1HtSDS) was clinically significantly higher in ≥2y group than in the control group (1.54 versus 1.16). In each group, there was significant difference between △2HtSDS and △3HtSDS. (Table 2; Fig. 1 and Supp. Figure 1).

**Table 2:** Comparisons of baseline height SDS, FAH SDS, and TH SDS

Treatment course	n	Baseline age (y)	Baseline height SDS	FAH SDS	TH SDS	△1HtSDS = FAH SDS - baseline height SDS	△2 HtSDS = FAH SDS - TH SDS	△3 HtSDS = baseline height SDS - TH SDS	Within different treatment courses Comparison between ∆2 and ∆3
< 3 m Control	41	12.9 ± 1.75	−3.14 ± 0.723	− 1.98 ± 1.065	−0.95 ± 0.749	1.16 ± 1.036	− 0.98 ± 1.106	− 2.22 ± 1.030	t = 4.927 P = 0.000*
3-6 m	70	12.7 ± 1.76	− 2.99 ± 0.904	− 1.68 ± 1.035	− 0.96 ± 0.720	1.30 ± 1.090 t = − 0.673 *P* = 0.503	− 0.78 ± 1.171	− 1.99 ± 0.997	t = 6.044 P = 0.000*
6-12 m	55	12.5 ± 1.86	− 2.87 ± 0.838	− 1.88 ± 1.196	− 0.99 ± 0.739	1.00 ± 1.273 t = 0.690 *P* = 0.492	− 0.94 ± 1.293	− 1.89 ± 0.900	t = 4.351 P = 0.000*
1–2y	81	12.3 ± 1.75	−2.79 ± 0.775	− 1.76 ± 1.417	− 0.93 ± 0.758	1.01 ± 1.312 t = 0.637 *P* = 0.525	− 0.84 ± 1.483	−1.88 ± 1.088	t = 4.920 P = 0.000*
≥2y	97	11.9 ± 1.89	−3.03 ± 1.268	− 1.49 ± 1.225	− 0.72 ± 0.778	1.54 ± 1.229 t = − 1.751 *P* = 0.082	−0.77 ± 1.475	−2.25 ± 1.560	t = 6.441 P = 0.000*
Total	344		−2.96 ± 0.968	− 1.72 ± 1.219	−0.89 ± 0.756	1.23 ± 1.222	−0.85 ± 1.349	− 2.05 ± 1.194	t = 11.764 *P* = 0.000*
		F = 3.693 *P* = 0.006*	F = 1.226 *P* = 0.299	F = 1.581 *P* = 0.179	F = 1.608 *P* = 0.172	F = 2.910 P = 0.022*	F = 0.261 *P* = 0.903	F = 1.500 *P* = 0.202

### Comparisons of FAH SDS and TH SDS

According to the Chinese Children Growth Standards, an SDS value of 1 is considered a clinically acceptable boundary value with practical clinical significance. Based on the gender stratification, the difference between FAH SDS and TH SDS was compared. The FAH SDS (− 1.913) was 1.079 lower than the TH SDS (− 0.834) (95% confidence interval [CI]: − 1.279 to − 0.878, *P* > 0.05) in boys and 0.398 lower (95% CI: − 0.625 to − 0.170, *P* < 0.001) in girls. Thus, the FAH SDS was closer to the TH SDS in girls after treatment.

### Analysis of patients with FAH attaining normal height

In total, 218 patients (218/344, 63.4%) had a FAH SDS > − 2 SD, attaining the normal and non-short height (Table 3), comprising 118 boys (118/217, 54.4%) and 100 girls (100/127, 78.7%). Among these patients, girls in the 1-2y group and ≥ 2y group had a FAH SDS higher than TH SDS. FAH SDS was lower than TH SDS in all groups of boys who attained the normal and non-short height (Table 3 and Supp. Figure 2).

**Table 3:** Comparisons of FAH SDS and TH SDS in the pooled group with FAH SDS > − 2 SD

Treatment course	Total					Girl					Boy				
	n	FAH SDS	TH SDS	t	P	n	FAH SDS	TH SDS	t	P	n	FAH SDS	TH SDS	t	P
< 3 m	19	−1.17 ± 0.741	−0.70 ± 0.585	−2.055	0.048	8	−0.81 ± 0.971	− 0.86 ± 0.656	0.118	0.908	11	−1.42 ± 0.429	−0.57 ± 0.525	−3.244	0.012
3–6 m	51	−1.20 ± 0.632	−0.93 ± 0.638	−1.988	0.050	21	−1.38 ± 0.543	−1.05 ± 0.815	− 1.471	0.150	30	−1.09 ± 0.612	−0.86 ± 0.516	− 1.649	0.112
6–12 m	31	−1.03 ± 0.676	−0.96 ± 0.859	−0.338	0.737	17	−1.01 ± 0.793	−0.97 ± 0.620	−0.144	0.886	14	−1.03 ± 0.547	−0.95 ± 1.100	−0.277	0.786
1–2 y	52	−0.88 ± 0.706	−0.88 ± 0.630	− 0.028	0.978	26	−0.99 ± 0.704	−1.08 ± 0.606	0.494	0.623	26	−0.83 ± 0.707	−0.66 ± 0.590	− 0.945	0.355
≥2 y	65	−0.76 ± 0.641	−0.71 ± 0.798	− 0.382	0.703	28	−0.61 ± 0.550	− 0.77 ± 0.917	0.744	0.461	37	−0.88 ± 0.713	−0.67 ± 0.709	−1.036	0.308

Compared with the pooled group with a FAH attaining the normal height, 8 of 10 girls and 11 of 31 boys in the control group attained the normal adult height. There was statistical significance between the number of boys in the control group and that in the pooled group (*P* = 0.049); that is, the number of boys in the control group attaining the normal height was smaller than that of boys in the pooled group reaching the normal height, while there was no significant difference between the control group and the pooled group.

### Analysis of influencing factors of FAH

A multivariate linear regression model was used to analyze the results, with FAH SDS as the dependent variable and the baseline age, baseline bone age, baseline height SDS, treatment course, and TH as independent variables. It was found that treatment course and baseline height SDS in the boys’ model were statistically significant (*P* < 0.05), whereas the baseline height SDS and baseline bone age affected the girls’ FAH SDS (Table 4).

**Table 4:** Analysis of influencing factors of FAH SDS

	Boys			Girls		
	Standardized coefficients	t	P	Standardized coefficients	t	P
(constant)		−2.862	0.005		2.063	0.042
FAH SDS	0.024	0.329	0.743	0.086	1.091	0.278
Baseline age	0.107	1.000	0.319	0.121	1.090	0.278
Baseline bone age (years)	0.090	0.893	0.373	−0.295	−2.810	0.006*
Baseline height standard deviation score (HtSDS)	0.356	4.371	0.000*	0.618	7.541	0.000*
Treatment course	0.158	2.136	0.034*	0.056	0.682	0.497

## Discussion

This is the first observational multicenter study with FAH results in China on the efficacy of growth hormone alone for ISS. It was an observational study based on the clinical reality and reflected the real-world situation. In addition to the follow-up of FAH, the difference between FAH SDS and baseline height SDS and between FAH height SDS and TH SDS was also evaluated. It was found that a treatment course of 2 years or more had better efficacy. Even for over 10 years old ISS children, GH therapy could improve FAH if the treatment course was long enough.

Randomized controlled trials (RCTs) have been recognized as high-quality research evidence because of their rigorous grouping/screening criteria and standardized treatment. However, sometimes RCTs do not actually reflect the clinical reality because the efficacy of GH for ISS varies greatly and the GH treatment is highly individualized in the true clinical setting. Although real-world retrospective studies are feasible, their data are limited by the natural characteristics of the treated children. In the study of short stature, it is difficult to find a control group without treatment: in clinical practice, few children will adhere to the follow-up protocol if no GH treatment is applied. In the current study, patients who had received GH treatment for less than 3 months were included in the control group, which was based on the following consideration: although GH is effective for a patient, such efficacy within a short period (3 months) can be neglected for the FAH; thus, the result of a short treatment course of up to 3 months is similar to that of natural growth. Our data comprised real-world clinical data, which may be used as evidence for long-term treatment of GH for ISS.

As known, the efficacy of GH in the treatment of ISS remains controversial. In a variety of prospective/retrospective, randomized/non-randomized, and controlled/non-controlled trials, some researchers concluded that GH treatment improved ISS while others considered it ineffective. The efficacies in these studies varied dramatically [12–18]. Some meta-analyses showed that GH-treated ISS patients might gain more FAH than untreated children, but were still shorter than the normal populations. In the meta-analysis performed by Deodati and Canfarani, three RCTs that followed the ISS patients until FAH were included, among which the average treatment course was 4.6–6.2 years [8]. In these three RCTs the treated group gained 0.79 SDS (4.7 cm) more height than the control group without GH treatment. In the largest trial, the height attainment was 0.8 SDS (5 cm) more in the treatment group than in the control group. In the study by Sotos and Tokar, the height attainment was 1.9 SDS in the treatment group and only 0.49 SDS in the untreated control group [3]. Wit et al. evaluated 239 children treated with GH, of whom only 50 were followed up until FAH. The FAH increased by 1.52–1.85 SDS compared with baseline height [10]. In our current study, the FAH of boys and girls increased by 1.16 SD and 1.36 SD compared with baseline height, which is basically consistent with results reported in the literature. Compared with the 6 m-2y group, the ≥2y group had significantly more FAH gain over the baseline height. Therefore, GH treatment for more than 2 years can achieve better efficacy in ISS patients.

Our study showed that 54.4% of boys and 78.7% of girls had a FAH SDS > −2SD after GH treatment. This result explains that GH therapy is effective even in peri-puberty children. Among the girls who had been treated with GH > 2 years, their FAH was higher than TH. This further confirmed that GH therapy could improve FAH and longer the better. For boys, FAH SDS was lower than TH SDS in all groups although they attained the normal height. It may be that boys need to improve their height longer than girls. In the GeNeSIS observation study by Pfäffle and colleagues, including United States, Germany and France data, shows that most children achieved near adult height (NAH) within the normal range (height SDS > − 2) after GH treatment [19]. The main population of our study is Chinese Han children. It shows that the same results as in Europe and America, the efficacy of growth hormone is similar.

The average age of boys and girls at baseline was 12.7 ± 1.87 years and 11.7 ± 11.7 years, with bone age of 11.7 ± 2.32 years and 10.1 ± 10.02 years, respectively. The relatively late initiation of treatment reflected the real-world situation. Multivariate linear regression model analysis showed that FAH is influenced positively by baseline height SDS in the boys and girls, whereas negatively by baseline bone age in girls, which was consistent with previous findings [1, 3, 8, 13, 20]. Many previous studies have demonstrated that the age when starting GH treatment is an important factor for its efficacy [1, 2, 6, 10, 20]. In this regard, our patients might have benefited more from GH therapy if the treatment had started earlier.

And we notice in this study, even in the control group, there is a spontaneous height gain of 1.16 SD. Economic factors need to be taken into account in the treatment of ISS. In China, growth hormone therapy for ISS is covered by families themselves. Whether to treatment or not, as well as the course of treatment, is decided by parents after weighing the family economic and height expectations.

This study had some limitations: Although it was a multi-center observation study, 344 of 475 cases entered the final analysis. It reflects “real-world” clinical practice and can be considered reliable, but which is not represented in random clinical trials. The sample is not big enough when stratification by treatment duration, if there is a larger sample in each subgroup, it will be better to validate our findings.

## Conclusions

In summary, both girls and boys of ISS improved FAH by GH therapy even if treatments begin over 10 years old and majority of them reached TH. Some peri-puberty ISS will have a spontaneous height gain. We recommend the course of GH treatment more than 2 years for girls, and longer courses for boys.

## Supplementary information

**Additional file 1: Supp. Figure 1.** △1HtSDS (i.e., FAH SDS minus baseline height SDS) in each group.

**Additional file 2: Supp. Figure 2.** Comparisons of FAH SDS and TH SDS among girls in the pooled group with FAH SDS > − 2 SD.

## Data Availability

The datasets generated and analyzed during the present study are available from the corresponding author on reasonable request.

## Abbreviations

ISS: Idiopathic short status
SD: Standard deviations
GH: Growth hormone
FAH: Final adult height
GnRHa: Gonadotropin-releasing hormone agonist
SDS: Standard deviation score
TH: Target height
HtSDS: Height standard deviation score
CI: Confidence interval
RCTs: Randomized controlled Trials
