# Supplementary figures and images for: Final adult height of children with idiopathic short stature: a multicenter study on GH therapy alone started during peri-puberty
Source: BMC Pediatr. 2020 Mar 28;20:138. doi: 10.1186/s12887-020-02034-8 (PMC7102429; doi:10.1186/s12887-020-02034-8)

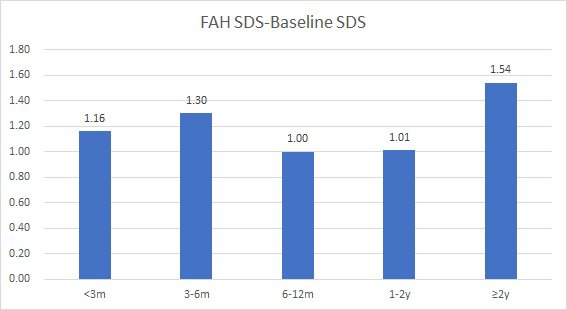

Supplement: Supplementary file 1 — Additional file 1: Supp. Figure 1. △1HtSDS (i.e., FAH SDS minus baseline height SDS) in each group. [file 12887_2020_2034_MOESM1_ESM.tif]

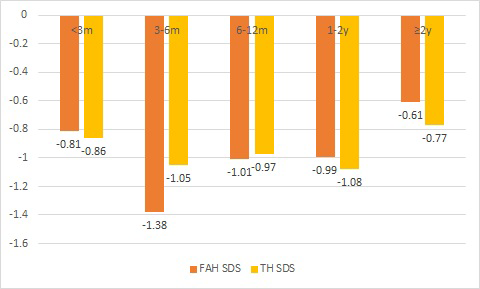

Supplement: Supplementary file 2 — Additional file 2: Supp. Figure 2. Comparisons of FAH SDS and TH SDS among girls in the pooled group with FAH SDS > − 2 SD. [file 12887_2020_2034_MOESM2_ESM.tif]
